# Evaluation of Physiological Characteristics, Soluble Sugars, Organic Acids and Volatile Compounds in ‘Orin’ Apples (*Malus domestica*) at Different Ripening Stages

**DOI:** 10.3390/molecules26040807

**Published:** 2021-02-04

**Authors:** Shunbo Yang, Zhipeng Meng, Yanan Li, Rongxin Chen, Yazhou Yang, Zhengyang Zhao

**Affiliations:** 1 College of Horticulture, Northwest Agricultural & Forestry University, Yangling 712100, China; yangshunboa@163.com (S.Y.); 15035600947@163.com (Z.M.); 15832496304@163.com (Y.L.); 15513999581@189.cn (R.C.); 15389084469@126.com (Y.Y.); 2 Apple Engineering and Technology Research Center of Shaanxi Province, Yangling 712100, China

**Keywords:** Orin, apple, fruit quality, HPLC, GC-MS, OAVs, flavor characteristics

## Abstract

‘Orin’ is a popular apple cultivar, which has a yellow-green appearance, pleasant taste, and unique aroma. However, few studies on the fruit quality characteristics of ‘Orin’ apples have been reported before. In this study, changes of the physiological characteristics were measured at different ripening stages, and the soluble sugars and organic acids were determined by high-performance liquid chromatography (HPLC). Volatile compounds were identified using the headspace solid-phase microextraction (HS-SPME) coupled with gas chromatography-mass spectrometry (GC-MS). During the fruit ripening, the ‘Orin’ apple fruit weight, size, and total soluble solid were gradually increased by contrast with the titratable acidity, and the firmness decreased. The content of four soluble sugars reached the maximum at the 180 days after full bloom (DAFB) stage. Malic acid was measured as the most abundant organic acid in ‘Orin’ apples. Ethyl butyrate, hexyl propanoate, hexyl acetate and butyl acetate belonging to esters with high odor activity values (OAVs) could be responsible for the typical aroma of ‘Orin’ apples. The aim of this work was to provide information on the flavor characteristics of ‘Orin’ apples and promote this apple cultivar for marketing and processing in the future.

## 1. Introduction

Apples (*Malus* × *domestica* Borkh.) are one of the most popular fruits and widely planted around the world. The quality of apples is influenced by attributes such as appearance, firmness, and flavor [1]. Flavor, as a key driver of consumer appreciation, determined by taste and aroma, is the most important and distinctive feature of apples [2,3]. Soluble sugars and organic acids directly affect the taste of apple fruits. Soluble sugars are responsible for the sweetness of apple fruits, and organic acids determine the sourness [4]. The major soluble sugars in apples are fructose, glucose, sucrose, and sorbitol, while the malic acid, citric acid, and tartaric acid mostly account for the organic acids [5]. The content of soluble sugars and organic acids in apples vary among varieties and some other factors such as light, exposure to sun, and other environmental factors could have an impact on it. Moreover, the accumulation of sugars and acids are diverse at different ripening stages. During the apple development, the synthesis and accumulation of soluble sugars are faster than that of organic acids [6]. Due to the significantly higher sugars/acid ratio, the taste of apple fruits at the ripe stage is much better than the immature fruits [7]. Additionally, a moderate concentration of organic acids will increase the fruit palatability and quality of apples [6,7].

Aroma, as an another important indicator of apple fruit quality, has a great impact on the overall flavor. Aroma is a complex mixture of volatile compounds and specific to the variety [8]. More than 300 volatile compounds have been identified in apples, including esters, alcohols, aldehydes, ketones, acids, and others [9,10]. During the growth of apple fruit, the profile of volatile compounds changes with the maturation. Aldehydes are the predominant volatile components in the early stage, then the content of alcohols begins to increase considerably, and finally esters play a dominant role in the profile of apple volatile compounds [11,12]. It was reported that apples contained more than 25 types of aldehydes, mostly hexanal, (E)-2-hexenal, and butanal [13]. Alcohols are formed by the reduction of corresponding aldehydes, the most abundant being 1-butanol, 2-methyl-1-butanol, and 1-hexanol [14]. Esters are the most important volatile compounds that contribute to the aroma of ripe apples. Due to the high content, butyl acetate, hexyl acetate, and 2-methylbutyl acetate have been reported as the crucial esters in apple fruits [15]. All these volatile compounds are greatly important for the complete characteristic aroma profile of apples.

‘Orin’ is a hybrid of the ‘Golden Delicious’ and ‘Indo’ apple cultivar, which has a yellow-green appearance, pleasant taste, and unique aroma. ‘Orin’ apples are widely planted in mang regions of China and are becoming more popular due to the high fruit quality. Most studies on the aroma volatiles of apple fruits have focused on the several varieties such as ‘Fuji’, ‘Jonagold’, and ‘Golden Delicious’ [16,17,18]. However, little is known about the flavor characteristics of ‘Orin’ apples, especially at different ripening stages. In this study, we evaluated the physiological characteristics, soluble sugars, organic acids, and aroma volatile compounds in ‘Orin’ apples at five different ripening stages. The aim of this work was to investigate the developmental physiological characteristics, soluble sugars, organic acids, and volatile compounds of the ‘Orin’ apple and provide valuable information for the fresh-eating and processing of this cultivar.

## 2. Results and Discussion

### 2.1. Changes of Physiological Characteristics

**Figure 1:**
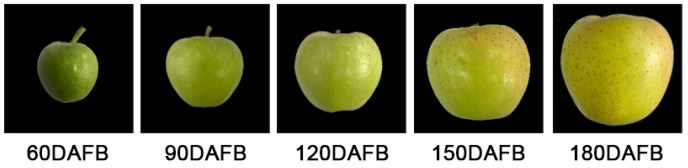
Color development in ‘Orin’ apple skin from 60–180 DAFB.

The basic fruit quality attributes of ‘Orin’ apples at different ripening stages were measured. As shown in Table 1, the changes of fresh weight, height, and diameter showed significant differences at different days after full bloom (DAFB). During fruit development, the weight of ‘Orin’ apples changed from 44.49 g at 60 DAFB to 292.80 g at 180 DAFB. At the same time, the apple fruit size was also increasing and reached the largest (height: 76.70 mm, diameter: 85.58 mm) at full ripening stage of 180 DAFB. The skin color of apple fruits is the most important indicator to evaluate ripeness [19]. Color perception is the result of three parameters L* (lightness), a* (red–green), and b* (yellow–blue) [20]. In this study, changes in ‘Orin’ skin color were visible at different ripening stages (Figure 1). From 60 DAFB to 180 DAFB, the values of color parameters L*, a*, and b* were continuously increasing with the fruit maturation. During the 60–90 DAFB stage, the values of L* and b* showed significant differences and changed quickly from 57.04 to 64.31 and from 31.99 to 38.35, respectively. While the values of a* were significantly increased from −18.64 to −13.67 during the 120–180 DAFB stage.

The total soluble solid (TSS) showed an upward trend during ‘Orin’ fruit ripening, which was consistent with the findings obtained on ‘Pink Lady’ apples in the previous study [21]. From 60 DAFB to 180 DAFB, the TSS significantly increased from 10.30 to 14.53 °Brix. On the contrary, the titratable acidity (TA) of ‘Orin’ fruits significantly decreased with respect to the ripening stages from 0.27–0.72%. At the stage of 180 DAFB, the TA in ‘Orin’ apples was 0.27%, and it was much lower than that at the other stages. The sugar acid ratio (TSS/TA) was increasing at 60–180 DAFB stages and ranged from 14.35 to 55.32. In addition, firmness is another important index for evaluating fruit maturity [22]. Fruit firmness of ‘Orin’ apples showed a downward trend from 19.45 kg/cm^2^ at the 60 DAFB stage to 6.27 kg/cm^2^ at the 180 DAFB stage, indicating the softening of the fruit throughout the ripening process.

### 2.2. Changes of Soluble Sugars and Organic Acids Content

Taste is the main characteristic of fruit quality and has a great impact on the consumer acceptance and purchase decision [23,24]. Sweet and sour are the most important tastes in apple fruits, which are determined by the composition and content of soluble sugars and organic acids [25]. In this study, four types of sugars including fructose, sucrose, glucose, and sorbitol in ‘Orin’ apples were measured by HPLC (Table 2). The content of four soluble sugars in ‘Orin’ apples generally increased during the fruit ripening periods and reached the maximum at full ripening stage of 180 DAFB. From 60 DAFB to 180 DAFB, the content of fructose significantly increased from 28.75 mg/g FW to 55.24 mg/g FW. At the 60 DAFB stage, the content of sucrose (4.70 mg/g FW) was lower than that of glucose (7.84 mg/g FW). With the fruit ripening, the content of sucrose increased to 20.93 mg/g FW at the 150 DAFB stage, higher than the 17.39 mg/g FW of glucose at the same stage. Moreover, the content of sorbitol increased slowly from 2.03 mg/g FW at the 60 DAFB stage to 2.44 mg/g FW at the 120 DAFB stage. After that, it increased rapidly from 2.44 mg/g FW to 6.07 mg/g FW during the 120–180 DAFB.

Malic acid was measured as the most abundant organic acid in ‘Orin’ apples, which was consistent with the previous studies [6,7,26]. Besides, citric acid and tartaric acid were detected in ‘Orin’ apples and their contents were much lower than the malic acid. During 60–180 DAFB, the content of malic acid decreased from 6.90 mg/g FW to 2.81 mg/g FW. The contents of citric acid and tartaric acid shared the similar changing trends with that of malic acid, which decreased from 0.10 mg/g FW to 0.05 mg/g FW and from 0.09 mg/g FW to 0.04 mg/g FW, respectively. Overall, the appropriate levels of sugar and acid are the important reasons for the ‘Orin’ apple flavor acceptability.

### 2.3. Volatile Compounds of Peel in ‘Orin’ Apple

Aroma is a complex mixture of volatile compounds, the composition and content of aroma volatiles showed different patterns during the fruit ripening [27]. In this study, a total of 47 volatile compounds were identified in ‘Orin’ apple peel, including 29 esters, 8 aldehydes, 3 alcohols, and 7 others (Table 3). At the 60 DAFB stage, 16 kinds of volatile compounds were detected in the peel of ‘Orin’ apples. Aldehydes were the main compounds, accounting for 95.09% of the total aroma volatiles (Table 4), which was in accordance with a previous study that reported that aldehydes were abundant at the early development stage of apples [28]. The content of (E)-2-hexenal and hexanal was up to 5669.86 µg/kg FW and 431.02 µg/kg FW, respectively. By contrast, the content of hexyl acetate and hexyl 2-methylbutyrate was only 34.41 µg/kg FW and 8.83 µg/kg FW, respectively. Similarly, aldehydes were the predominant volatile compounds at the 90 DAFB stage, which accounted for 96.17% of the total content. At the 120 DAFB stage, the content of esters was just slightly increased with the aldehydes decreased. Compared to the 60 DAFB stage, the content of 2-hexenal largely decreased to 1913.52 µg/kg FW and the content of hexyl acetate increased to 80.68 µg/kg FW at the 120 DAFB stage. At the 150 DAFB stage, 31 kinds of volatile compounds were observed in the peel of ‘Orin’ apple. A large number of volatile compounds belonging to the group of esters such as butyl acetate, butyl 2-methylbutyrate, and hexyl 2-methylbutyrate were generated. As a result, the level of esters was increased dramatically, which contributed to 67.65% of the total volatiles content. At the 180 DAFB stage, there were 33 volatile compounds identified in the ‘Orin’ peel, and esters were the most abundant volatiles, accounting for 70.40%, in comparison with aldehydes, only accounting for 7.90%, which was consistent with the previous study [29]. Moreover, the content of hexyl 2-methylbutyrate, hexyl butyrate, and butyl caproate showed high levels at the 180 DAFB stage, which were 8164.16 µg/kg FW, 5505.92 µg/kg FW, and 1054.14 µg/kg FW, respectively. These results indicated that the typical aromatic compounds in ‘Orin’ apple peel were the fruity esters and reached the maximum at the full ripening of 180 DAFB.

### 2.4. Volatile Compounds of Pulp in ‘Orin’ Apple

A total of 32 volatiles were identified in the ‘Orin’ pulp, including 18 esters, 7 aldehydes, 4 alcohols, and 3 other compounds (Table 5). During the 60–120 DAFB, alcohols were the major group of volatile compounds contributing to apple aroma in ‘Orin’ apple pulps, accounting for over 84.11% (Table 6). In accordance with the previous study, 1-hexanol were the most dominant alcohols identified in ‘Orin’ apples [30]. The content of 1-hexanol decreased from 305.32 µg/kg FW at the 60 DAFB stage to 81.63 µg/kg FW at the 150 DAFB stage, then increased to 132.18 µg/kg FW at the 180 DAFB stage, playing a dominant role in aroma volatiles of ‘Orin’ apple pulps. In addition, aldehydes confer green odors to apples [29], the content of (Z)-2-heptenal continuously decreased from 34.97 µg/kg FW to 16.61 µg/kg FW during the 60–150 DAFB. Generally, esters provide fruity notes and contribute to the characteristic aroma of apple fruits [31]. During the 150–180 DAFB, the content of esters had the largest proportions, which was in accordance with a previous report on aromatic profile of ‘Fuji’ apples [32]. At the 180 DAFB stage, there were 12 esters among the 24 volatile compounds, accounting for 58.77% of the total volatiles. The content of butyl acetate (217.52 µg/kg FW), hexyl propanoate (395.66 µg/kg FW), and butyl caproate (399.42 µg/kg FW) in pulps showed high levels at the 180 DAFB full-ripening stage. The content of esters increased with the aldehydes decreased during the ‘Orin’ apple ripening. From 120 DAFB to 150 DAFB, the varieties of volatiles increased rapidly from 7 to 25, especially the kinds of esters increased from the number 4 to 13, suggesting that this stage was a critical period for the formation of aroma compounds in ‘Orin’ apple pulps.

### 2.5. Characteristic Aroma Profile for Peel and Pulp in ‘Orin’ Apples

In order to evaluate the contribution of volatile compounds to the aroma in ‘Orin’ apple fruits, the ratio between the concentration of compound and the perception threshold, which known as the odor activity value (OAV), was calculated in ‘Orin’ peels and pulps at the 180 DAFB (full ripening) stage. The higher the OAV, the greater the contribution of the volatile compounds made to the whole aroma profile [33,34]. As shown in Table 7, a total of 17 volatile compounds were identified as the effective compounds (OAV > 1) on the basis of OAV analysis. The 12 esters with high OAVs in peels of ‘Orin’ apples were ethyl butyrate, ethyl 2-methylbutyrate, butyl acetate, 2-methylbutyl acetate, butyl butyrate, butyl 2-methylbutyrate, hexyl acetate, pentyl butyrate, hexyl propanoate, hexyl butyrate, hexyl 2-methylbutyrate, and hexyl hexanoate. Moreover, 4 compounds (2 aldehydes, 1 alcohol, and 1 other compound) with high OAVs were hexanal, 2-hexenal, 1-hexanol, and estragole. In comparison, there are only 5 volatile compounds with the OAVs greater than 1 in the pulps of ‘Orin’ apple. The high OAVs were exhibited by ethyl butyrate, hexyl propanoate, hexyl acetate, butyl acetate, and 3-hexenal, indicating these compounds contributed greatly to the aroma in the ‘Orin’ apple pulps. In general, there were more effective volatile compounds in peels than that in pulps, which was consistent with previous study reported that volatile compounds in apples were primarily synthesized in the fruit skin [35]. The volatiles substances of esters were previously identified as the most important aroma contributors in apples [36]. In this study, four volatile compounds (ethyl butyrate, hexyl propanoate, hexyl acetate, butyl acetate) belonging to esters with high OAVs (OAV > 1) were detected both in peels and pulps and might be responsible for the typical aroma of ‘Orin’ apples.

## 3. Materials and Methods

### 3.1. Plant Materials

The trees of ‘Orin’ apples were planted in 2010 at the experimental station of Northwest A&F University, Baishui County, Shaanxi Province, China (35°21′ N, 109°55′ E). ‘Orin’ apples were collected every 30 days from 60 days after full bloom (DAFB) until fully ripening 180 DAFB in 2019. Three biological replicates from three trees were prepared, with 4–6 fruits per replicate. Whole fruits were used for color measurements and firmness tests. After that, apple peel and pulp were carefully dissected. All samples were immediately frozen in liquid nitrogen and stored at −80 °C until extraction.

### 3.2. Measurement of Physiological Characteristics

Fruit height and diameter were measured by a digital vernier caliper (Meinaite, Chengdu, China). Single fruit weight was determined using an electronic balance (Mettler-Toledo Inc., Greifensee, Switzerland). A Chroma Meter CR-400 chromaportable colorimeter (Konica Minolta, Tokyo, Japan) was used to measure the color of apple peels. Fruit chromaticity was recorded using the Comission Internationale de l’ Eclairage (CIE) parameters L*, a*, and b*. A texture analyzer (FTA GS-15, Berlin, Germany; test depth 8 mm) equipped with a 10 mm diameter flat probe was used for the determination of flesh firmness. The fruit total soluble solid (TSS) and titratable acidity (TA) was measured by a hand refractometer (Atago, Tokyo, Japan) and a digital fruit acidity meter (GMK-835F Perfect, Berlin, Germany), respectively.

### 3.3. Determination of Sugars and Organic Acids

According to the method described by Ma et al. [3] with some modifications, soluble sugar contents were measured by the high-performance liquid chromatography (HPLC). The samples of apple flesh (5.00 g) were crushed by a cell grinder (BILON96-II, Shanghai, China) and diluted to 25 mL with redistilled water, ultrasonically extracted at 80 °C for 60 min. Then, the suspension was centrifuged at 10,000× *g* for 20 min and cooled to room temperature. The supernatant was subsequently collected and filtered into a vial through 0.45 μm Millipore membrane filters (Millipore Corporation, Bedford, OH, USA). The content of sugars and organic acids were analyzed by HPLC (Waters, Milford, CT, USA). The separation of soluble sugars was performed by a Sugar Pak TM I column from Waters (300 mm × 6.5 mm) operated at 80 °C with the injection volume of 20 μL. Bi-distilled water was used as the mobile phase at the flow rate 0.6 mL/min. Each run was 25 min and the content of sugars (fructose, glucose, sucrose, and sorbitol) were calculated by using external standards.

The extractions used for organic acids analysis was the same with the soluble sugars analysis. According to the method described by Liu et al. [39], HPLC with an IC PAK TM ION exclusion column (300 mm × 7.8 mm) (Waters, Milford, CT, USA) and a PDA detector set at 210 nm were used for the organic acids analysis. The temperature of column was 40 °C and the elution solvent was 0.01 mM sulfuric acid in bi-distilled water, whose flow rate was 0.5 mL/min. The analysis lasted 30 min and the results were expressed as mg/g.

### 3.4. Determination of Volatile Compound

The volatile compounds were extracted using the headspace solid-phase microextraction (HS-SPME). All extractions were performed by a divinylbenzene /carboxen/polydi-methylsiloxane (DVB/CAR/PDMS) fibre, of 50/30 µm thickness (Supelco, Bellefonte, PA, USA). For volatile analysis, 5 g apple samples were placed into a 50 mL screw-cap headspace vial containing a magnetic stirring rotor and 1g NaCl spiked with 10 μL (0.4 mg/mL) 3-nonanone (internal standard). After the headspace bottle equilibrated at 50 °C for 10 min on a metal heating agitation platform, the SPME fiber was inserted into the headspace with continuous heating and agitation (200 rpm) for 30 min to adsorb volatile substances. Finally, it was introduced into the heated injector port of the chromatograph for desorption at 250 °C for 2.5 min.

Analysis of volatile compounds in apple fruit was performed by a Thermo Trace GC Ultra gas chromatograph (Agilent Technologies Inc., Palo Alto, CA, USA), equipped with a HP-INNOWax capillary column (60 m × 0.25 mm × 0.25 μm). The oven temperature was programmed as follows: 40 °C held for 3 min, rising to 150 °C at 5 °C/min, then increased at 10 °C/min to 220 °C and held for 5 min. Helium was circulated as the carrier gas at a constant flow rate of 1.0 mL/min in splitless mode. Both the temperature of ion source and transfer line were maintained at 240 °C. The MS fragmentation was performed under an electron ionization of 70 eV with a scan range of 35–450 *m/z*.

Xcalibur 3.2 software was used to process the data collected from the GC-MS. Linear retention indices were calculated under the same chromatographic conditions after the injection of a C7-C30 n-alkane series (Supelco, Bellefonte, PA, USA). Identification of volatile compounds was assigned by the comparison of retention indices (RI) and the database of NIST/EPA/NIH Mass Spectral Library (NIST 2014, https://www.nist.gov/srd). Based on the total ion chromatogram, the content of each volatile was quantified as 3-nonanone equivalent (internal standard) by the peak area.

### 3.5. Statistical Analysis

The data were expressed as mean value ± standard deviation of at least three replicates. Statistical analysis and one-way analysis of variance (ANOVA) were performed by SPSS Version 19.0 (SPSS Ins., Chicago, IL, USA), differences between the samples were estimated with the Duncan test (*p* < 0.05).

## 4. Conclusions

In this study, the physiological characteristics (weight, size, color, soluble solid, titratable acidity, and firmness), soluble sugars, organic acids, and volatile compounds in ‘Orin’ apples were determined at five different ripening stages. During the development, the apple fruit weight, size, and total soluble solid were gradually increased by contrast with the titratable acidity and firmness decreased. The content of four sugars (fructose, sucrose, glucose, and sorbitol) in ‘Orin’ apples generally increased during the fruit ripening period and reached the maximum at the full ripening stage of 180 DAFB. Malic acid was measured as the most abundant organic acid in ‘Orin’ apples. From 60 DAFB to 180 DAFB, the contents of citric acid and tartaric acid shared the similar changing trends with that of malic acid, which decreased with the fruit ripening. In this study, 47 and 32 volatile compounds were identified in ‘Orin’ apple peel and pulp, respectively. From 120 DAFB to 180 DAFB, the content of esters in peels was dramatically increased while the aldehydes decrease. Similarly, the content of esters in pulps had the largest proportions during the 150–180 DAFB. On the basis of OAV analysis, four compounds (ethyl butyrate, hexyl propanoate, hexyl acetate, butyl acetate) belonging to esters with high OAVs were detected both in peel and pulp at the 180 DAFB stage and could be responsible for the typical aroma of ‘Orin’ apples. This work could provide important information on the flavor characteristics of ‘Orin’ apples and might be valuable to promote this apple cultivar for the marketing and processing in the future.

## Figures and Tables

**Table 1:** Physiological characteristics of ‘Orin’ apple at different ripening stages.

	60 DAFB	90 DAFB	120 DAFB	150 DAFB	180 DAFB
Fresh weight (g)	44.49 ± 1.99^e^	138.08 ± 11.16^d^	172.35 ± 6.19^c^	255.16 ± 24.86^b^	292.80 ± 31.99^a^
Height (mm)	46.15 ± 1.64^c^	59.64 ± 1.78^b^	65.50 ± 1.45^b^	73.91 ± 5.75^a^	76.70 ± 7.54^a^
Diameter (mm)	44.99 ± 2.02^c^	67.47 ± 2.88^b^	68.63 ± 2.18^b^	81.52 ± 2.62^a^	85.58 ± 5.92^a^
L* value	57.04 ± 1.55^c^	64.31 ± 0.99^b^	66.67 ± 0.83^ab^	69.05 ± 1.71^a^	69.20 ± 2.66^a^
a* value	−19.51 ± 0.23^c^	−19.11 ± 0.97^c^	−18.64 ± 0.82^c^	−16.45 ± 0.96^b^	−13.67 ± 1.57^a^
b* value	31.99 ± 2.86^b^	38.35 ± 1.00^a^	39.15 ± 1.40^a^	39.87 ± 1.67^a^	40.11 ± 1.97^a^
TSS (°Brix)	10.30 ± 0.08^e^	11.05 ± 0.31^d^	11.58 ± 0.26^c^	13.70 ± 0.55^b^	14.53 ± 0.17^a^
TA (%)	0.72 ± 0.05^a^	0.66 ± 0.03^a^	0.47 ± 0.02^b^	0.35 ± 0.07^c^	0.27 ± 0.04^d^
TSS/TA ratio	14.35 ± 0.90^d^	16.83 ± 0.80^cd^	24.53 ± 0.95^c^	40.14 ± 8.53^b^	55.32 ± 9.07^a^
Firmness (kg/cm^2^)	19.45 ± 2.16^a^	13.63 ± 0.46^b^	9.60 ± 0.21^c^	8.40 ± 0.19^c^	6.27 ± 0.26^d^

Values are means ± standard deviation (*n* = 4). Different lowercase superscripts indicate significant difference by Duncan test at *p* < 0.05 level. DAFB—days after full bloom, TSS—total soluble solid, TA—titratable acidity.

**Table 2:** The content of soluble sugars and organic acids in ‘Orin’ apple at different ripening stages.

	60 DAFB	90 DAFB	120 DAFB	150 DAFB	180 DAFB
**Sugars** (mg/g FW)					
Fructose	28.75 ± 0.13^e^	41.33 ± 1.03^d^	49.22 ± 0.90^c^	53.28 ± 0.94^b^	55.24 ± 0.40^a^
Sucrose	4.70 ± 0.46^d^	6.08 ± 0.14^d^	9.51 ± 0.21^c^	20.93 ± 0.56^b^	32.07 ± 1.21^a^
Glucose	7.84 ± 0.26^e^	9.08 ± 0.14^d^	13.18 ± 0.21^c^	17.39 ± 0.16^b^	18.53 ± 0.12^a^
Sorbitol	2.03 ± 0.09^d^	2.12 ± 0.03^d^	2.44 ± 0.10^c^	5.50 ± 0.09^b^	6.07 ± 0.08^a^
**Acids** (mg/g FW)					
malic acid	6.90 ± 0.29^a^	5.42 ± 0.20^b^	4.74 ± 0.18^c^	3.56 ± 0.19^d^	2.81 ± 0.15^e^
citric acid	0.10 ± 0.01^a^	0.08 ± 0.01^b^	0.06 ± 0.02^c^	0.05 ± 0.01^c^	0.05 ± 0.01^c^
tartaric acid	0.09 ± 0.01^a^	0.09 ± 0.01^a^	0.08 ± 0.01^b^	0.04 ± 0.01^c^	0.04 ± 0.01^c^

Values are means ± standard deviation (*n* = 4). Different lowercase superscripts indicate significant difference by Duncan test at *p* < 0.05 level. DAFB—days after full bloom.

**Table 3:** The content (µg/kg FW) of volatile compounds in ‘Orin’ apple peels at different ripening stages.

Compounds	CAS	60 DAFB	90 DAFB	120 DAFB	150 DAFB	180 DAFB
**Esters**						
Ethyl butyrate	105-54-4	–	–	–	–	71.66
Ethyl 2-methylbutyrate	7452-79-1	–	–	–	–	11.93
Butyl acetate	123-86-4	–	–	–	68.26	115.64
2-Methylbutyl acetate	624-41-9	–	–	–	200.05	188.15
Butyl propionate	590-01-2	–	–	–	–	137.84
Amyl acetate	628-63-7	–	–	–	24.38	20.77
Butyl butyrate	109-21-7	–	–	–	190.18	972.18
Butyl 2-methylbutyrate	15706-73-7	–	–	–	406.10	992.24
2-Methylbutyl butyrate	51115-64-1	–	–	–	18.88	78.49
Hexyl acetate	142-92-7	34.41	7.20	80.68	534.73	925.04
2-Methylbutyl 2-methylbutyrate	2445-78-5	–	–	–	42.73	101.89
Pentyl butyrate	540-18-1	–	–	–	34.79	185.86
Amyl 2-methylbutyrate	68039-26-9	–	–	–	132.70	257.60
2-Hexenol acetate	10094-40-3	41.09	11.89	8.08	–	–
Hexyl propanoate	2445-76-3	–	–	–	47.82	422.72
Hexyl isobutyrate	2349-07-7	–	–	–	141.75	477.96
Butyl caproate	626-82-4	–	–	–	356.84	1054.14
Hexyl butyrate	2639-63-6	7.69	–	–	931.06	5505.92
Hexyl 2-methylbutyrate	10032-15-2	8.83	12.02	13.46	7767.45	8164.16
2-Methylbutyl hexanoate	2601-13-0	–	–	–	33.13	92.26
Amyl caproate	540-07-8	–	–	–	137.91	–
Heptyl 2-methylbutyrate	50862-12-9	–	–	–	118.31	271.34
Hexyl hexanoate	6378-65-0	11.77	–	–	2073.11	316.32
Hexyl tiglate	16930-96-4	–	–	–	143.10	–
Hexyl caprylate	1117-55-1	–	–	–	209.84	261.86
Octyl hexanoate	4887-30-3	–	–	11.27	–	–
Butyl caprylate	589-75-3	–	–	–	500.55	–
2-Pentyl octanoate	55193-30-1	–	–	–	109.59	–
Octyl heptanoate	5132-75-2	–	–	–	–	90.43
**Alcohols**						
2-Methyl-1-butanol	137-32-6	–	–	–	–	52.35
2-Hexyn-1-ol	764-60-3	76.98	53.97	27.67	58.26	20.66
1–Hexanol	111-27-3	–	–	–	55.22	171.25
**Aldehydes**						
Hexanal	66-25-1	431.02	454.14	159.33	127.30	158.79
2-Methyl-4-pentenal	5187-71-3	–	10.19	6.00	–	–
(E)-2-Hexenal	505-57-7	5669.86	4122.50	1913.52	2210.68	2164.99
3-Hexenal	4440-65-7	6.86	17.00	14.99	–	–
Nonanal	124-19-6	–	–	5.87	–	–
(E,E)-2,4-Heptadienal	4313-03-5	19.92	7.39	6.56	–	–
Benzaldehyde	100-52-7	80.79	–	–	–	–
(E,E)-2,4-Hexadienal	142-83-6	23.99	23.03	3.99	–	–
**Others**						
Dodecane	112-40-3	24.82	19.70	–	–	–
Tetradecane	629-59-4	70.00	44.82	5.59	–	39.85
Estragole	140-67-0	–	–	–	26.40	456.98
(E)-α-Bergamotene	13474-59-4	–	–	–	466.54	1131.35
α-Farnesene	502-61-4	15.10	11.67	75.14	3805.96	4435.00
Thujopsene	470-40-6	31.89	17.01	–	49.71	77.05
Copaene	3856-25-5	–	6.19	9.51	–	–

Volatile compounds identified by the methods: MS—mass spectra comparison with published data and MS library (NIST14); RI—retention index agreed with the data reported in previous papers or a database on the web (http://www.odour.org.uk/lriindex.html). Data are means of three replications. DAFB indicates days after full bloom. – indicates not detected.

**Table 4:** Total content (µg/kg) and percentage (%) of each type of volatiles in ‘Orin’ apple peels at different ripening stages.

Class	60 DAFB	90 DAFB	120 DAFB	150 DAFB	180 DAFB
Esters	103.78/1.58	31.10/0.65	113.49/4.85	14223.24/67.65	20716.42/70.40
Alcohols	76.98/1.17	53.97/1.12	27.67/1.18	113.48/0.55	244.27/0.83
Aldehydes	6232.44/95.09	4634.25/96.17	2110.26/90.12	2337.99/11.12	2323.78/7.90
Others	141.82/2.16	99.38/2.06	90.25/3.85	4348.60/20.68	6140.23/20.87

**Table 5:** The content (µg/kg FW) of volatile compounds in ‘Orin’ apple pulps at different ripening stages.

Compounds	CAS	60 DAFB	90 DAFB	120 DAFB	150 DAFB	180 DAFB
**Esters**						
Ethyl butyrate	105-54-4	–	–	–	13.00	68.43
Butyl acetate	123-86-4	1.11	3.26	4.28	84.23	217.52
Butyl propionate	590-01-2	–	–	–	1.36	5.79
Amyl acetate	628-63-7	–	–	–	1.72	7.52
Butyl butyrate	109-21-7	9.37	2.21	–	–	–
Hexyl acetate	142-92-7	–	–	–	1.97	11.85
2-Methylbutyl 2-methylbutyrate	2445-78-5	–	–	–	22.03	93.17
Amyl 2-methylbutyrate	68039-26-9	–	0.70	–	0.83	–
2-Hexenol acetate	10094-40-3	–	–	–	1.28	12.04
Hexyl propanoate	2445-76-3	–	–	–	47.25	395.66
Hexyl isobutyrate	2349-07-7	0.70	–	–	–	–
Butyl caproate	626-82-4	–	1.28	20.02	42.24	399.42
Hexyl butyrate	2639-63-6	–	0.72	0.92	1.18	3.21
2-Methylbutyl hexanoate	2601-13-0	1.39	0.84	6.00	–	–
Hexyl hexanoate	6378-65-0	–	0.89	–	–	–
Hexyl caprylate	1117-55-1	–	–	–	1.29	7.42
Octyl hexanoate	4887-30-3	8.70	1.19	–	–	–
Butyl caprylate	589-75-3	–	–	–	4.39	32.67
**Alcohols**						
1-Butanol	71-36-3	–	–	–	2.88	12.23
2-Methyl-1-butanol	137-32-6	4.15	1.99	2.42	–	–
2-Hexyn-1-ol	764-60-3	–	–	–	7.11	51.30
1-Hexanol	111-27-3	305.32	273.68	268.33	81.63	132.18
**Aldehydes**						
Hexanal	66-25-1	–	–	–	1.11	–
2-Methyl-4-pentenal	5187-71-3	–	–	–	5.47	8.18
(E)-2-Hexenal	505-57-7	–	–	–	3.40	30.24
3-Hexenal	4440-65-7	–	–	–	13.49	49.23
(Z)-2-Heptenal	57266-86-1	34.97	30.53	25.52	16.61	55.98
Nonanal	124-19-6	2.23	0.60	–	30.08	44.09
(E)-2-Octenal	2548-87-0	–	–	–	3.17	18.23
**Others**						
Tetradecane	629-59-4	–	0.89	–	2.67	141.76
(E)-α-Bergamotene	13474-59-4	–	–	–	–	9.37
α-Farnesene	502-61-4	–	–	–	21.72	327.40

Volatile compounds identified by the methods: MS—mass spectra comparison with published data and MS library (NIST14); RI—retention index agreed with the data reported in previous papers or a database on the web (http://www.odour.org.uk/lriindex.html). Data are means of three replications. DAFB indicates days after full bloom. – indicates not detected.

**Table 6:** Total content (µg/kg) and percentage (%) of each type of volatiles in ‘Orin’ apple pulps at different ripening stages.

Class	60 DAFB	90 DAFB	120 DAFB	150 DAFB	180 DAFB
Esters	21.27/5.78	11.10/3.48	31.23/9.53	222.78/54.05	1254.71/58.77
Alcohols	309.47/84.11	275.68/86.48	270.75/82.67	91.62/22.23	195.71/9.17
Aldehydes	37.20/10.11	31.13/9.76	25.52/7.80	73.35/17.80	205.95/9.65
Others	0.00/0.00	0.89/0.28	0.00/0.00	24.39/5.92	478.52/22.41

**Table 7:** Odor activity values of volatile compounds reaching a concentration above the odor threshold (OAV > 1) in ‘Orin’ apple peels and pulps at the 180 DAFB stage.

Compounds	Odor Descriptor	Odor Threshold * (μg/kg)	Peel	Pulp
Ethyl butyrate	Fruity	1	71.66	68.43
Ethyl 2-methylbutyrate	Fruity, green apple	0.006	1988.76	–
Butyl acetate	Fruity	66	1.75	3.30
2-Methylbutyl acetate	Characteristic apple, banana	5	37.63	–
Butyl butyrate	Rotten apple	100	9.72	–
Butyl 2-methylbutyrate	Fruity	17	58.37	–
Hexyl acetate	Sweet, fruity, floral	2	462.71	5.92
Pentyl butyrate	Fruity, apple, banana	59	3.15	–
Hexyl propanoate	Sweet, fruity,	8	52.84	49.46
Hexyl butyrate	Fruity, banana, pineapple	250	2.22	0.13
Hexyl 2-methylbutyrate	Fruity, apple	22	371.10	–
Hexyl hexanoate	Sweet, fruity	10	31.63	–
Hexanal	Green	5	31.76	–
(E)-2-Hexenal	Grass, herbaceous	17	127.35	0.20
3-Hexenal	Grass	0.25	–	196.93
1-Hexanol	Fresh, green, earthy	150	1.14	0.88
Estragole	Anise	7.5	60.93	–

* Odor descriptors and odor threshold were reported in the literature [10,36,37,38].

## Data Availability

The data presented in this study are available on request from the corresponding authors.

## Sample Availability

Samples of the ‘Orin’ apple fruits are available from the authors.

## References

[1] Espino-Diaz M., Sepulveda D.R., Gonzalez-Aguilar G., Olivas G.I. (2016). Biochemistry of apple aroma: A review. Food Technol. Biotech..

[2] Perez A.G., Sanz C., Bruckner B., Wyllie S.G. (2008). Formation of fruit flavour. Fruit and Vegetable Flavour.

[3] Ma B., Chen J., Zheng H., Fang T., Ogutu C., Li S., Han Y., Wu B. (2015). Comparative assessment of sugar and malic acid composition in cultivated and wild apples. Food Chem..

[4] Famiani F., Battistelli A., Moscatello S., Cruz-Castillo J.G., Walker R.P. (2015). The organic acids that are accumulated in the flesh of fruits: Occurrence, metabolism and factors affecting their contents-a review. Rev. Chapingo Ser. Hortic..

[5] Ma C., Sun Z., Chen C.B., Zhang L.L., Zhu S.H. (2014). Simultaneous separation and determination of fructose, sorbitol, glucose and sucrose in fruits by HPLC–ELSD. Food Chem..

[6] Zhang Y.Z., Li P.M., Cheng L.L. (2010). Developmental changes of carbohydrates, organic acids, amino acids, and phenolic compounds in ‘Honeycrisp’ apple flesh. Food Chem..

[7] Liu Y., Chen N., Ma Z., Che F., Mao J., Chen B. (2016). The changes in color, soluble sugars, organic acids, anthocyanins and aroma components in ‘starkrimson’ during the ripening period in china. Molecules.

[8] Aprea E., Corollaro M.L., Betta E., Endrizzi I., Dematte M.L., Biasioli F., Gasperi F. (2012). Sensory and instrumental profiling of 18 apple cultivars to investigate the relation between perceived quality and odour and flavour. Food Res. Int..

[9] Echeverria G., Fuentes T., Graell J., Lara I., Lopez M. (2004). Aroma volatile compounds of ‘Fuji’ apples in relation to harvest date and cold storage technology: A comparison of two seasons. Postharvest Biol. Technol..

[10] Guo J., Yue T., Yuan Y., Sun N., Liu P. (2020). Characterization of volatile and sensory profiles of apple juices to trace fruit origins and investigation of the relationship between the aroma properties and volatile constituents. LWT–Food Sci. Technol..

[11] Fellman J.K., Miller T.W., Matinson D.S., Matheis J.P. (2000). Factors that influence biosynthesis of volatile flavor compounds in apple fruits. Hort. Sci..

[12] Zhu Y., Rudell D.R., Mattheis J.P. (2008). Characterization of cultivar differences in alcohol acyltransferase and 1-aminocyclopropane-1-carboxylate synthase gene expression and volatile ester emission during apple fruit maturation and ripening. Postharvest Biol. Technol..

[13] Dimick P.S., Hoskin J.C., Acree T.E. (1983). Review of apple flavor–state of the art. Crit. Rev. Food Sci..

[14] Fellman J.K., Rudell D.R., Matinson D.S., Matheis J.P. (2003). Relationship of harvest maturity to flavor regeneration after CA storage of ‘Delicious’ apples. Postharvest Biol. Technol..

[15] Dunemann F., Ulrich D., Malysheva-Oto L., Weber W., Longhi S., Velasco R., Costa F. (2012). Functional allelic diversity of the apple alcohol acyl-transferase gene *MdAAT1* associated with fruit ester volatile contents in apple cultivars. Mol. Breed..

[16] Iglesias I., Echeverria G., Lopez M.L. (2012). Fruit color development, anthocyanin content, standard quality, volatile compound emissions and consumer acceptability of several ‘fuji’ apple strains. Sci. Hortic..

[17] Contreras C., Tjellstrom H., Beaudry R.M. (2015). Relationships between free and esterified fatty acids and lox-derived volatiles during ripening in apple. Postharvest Biol. Technol..

[18] Salas N.A., Gonzalez-Aguilar G.A., Jacobo-Cuellar J.L., Espino M., Sepulveda D., Guerrero V., Olivas G.I. (2016). Volatile compounds in golden delicious apple fruit (*Malus domestica*) during cold storage. Rev. Fitotec. Mex..

[19] Liu Y.L., Che F., Wang L.X., Meng R., Zhang X.J., Zhao Z.Y. (2013). Fruit coloration and anthocyanin biosynthesis in non-red and red apples (*Malus*×*domestica* Borkh.) after bag removal. Molecules.

[20] Jesus A.L.T., Leite T.S., Cristianini M. (2018). High isostatic pressure and thermal processing of açai fruit (*Euterpe oleracea Martius*): Effect on pulp color and inactivation of peroxidase and polyphenol oxidase. Food Res. Int..

[21] Villatoro C., Altisent R., Echeverria G., Graell J., Lara M.L.L. (2008). Changes in biosynthesis of aroma volatile compounds during on-tree maturation of ‘Pink Lady’ apples. Postharvest Biol. Technol..

[22] Zhu X., Li Q., Li J., Luo J., Chen W., Li X. (2018). Comparative study of volatile compounds in the fruit of two banana cultivars at different ripening stages. Molecules.

[23] Malundo T.M.M., Shewfelt R.L., Scott J.W. (1995). Flavor quality of fresh tomato (*Lycopersicon esculentum* Mill.) as affected by sugar and acid levels. Postharvest Biol. Technol..

[24] Roberts G., Spadafora N.D. (2020). Analysis of apple flavours: The use of volatile organic compounds to address cultivar differences and the correlation between consumer appreciation and aroma Profiling. J. Food Qual..

[25] Yoon H.K., Kleiber T., Zydlik Z., Rutkowski K., Morkunas I. (2020). A comparison of selected biochemical and physical characteristics and yielding of fruits in apple cultivars (*Malus domestica* borkh.). Agronomy.

[26] Hecke K., Herbinger K., Veberiac R., Trobec M., Toplak H., Stampar F., Keppel H., Grill D. (2006). Sugar-, acid- and phenol contents in apple cultivars from organic and integrated fruit cultivation. Eur. J. Clin. Nutr..

[27] Schiller D., Contreras C., Vogt J., Dunemann F., Defilippi B.G., Beaudry R., Schwab W. (2015). A dual positional specific lipoxygenase functions in the generation of flavor compounds during climacteric ripening of apple. Hort. Res..

[28] Vallat A., Gu H., Dorn S. (2005). How rainfall, relative humidity and temperature influence volatile emissions from apple trees in situ. Phytochemistry.

[29] Zhu D., Ren X., Wei L., Cao X., Ge Y., Li J. (2020). Collaborative analysis on difference of apple fruits flavour using electronic nose and electronic tongue. Sci. Hortic..

[30] Gan H.H., Soukoulis C., Fisk I. (2014). Atmospheric pressure chemical ionisation mass spectrometry analysis linked with chemometrics for food classification—A casestudy: Geographical provenance and cultivar classification of monovarietal clarified apple juices. Food Chem..

[31] Dixon J., Hewett E.W. (2000). Factors affecting apple aroma/flavour volatile concentration: A review. N. Z. J. Crop Hortic. Sci..

[32] Echeverria G., Graell J., Lopez M., Lara I. (2004). Volatile production, quality and aroma related enzyme activities during maturation of ‘Fuji’ apples. Postharvest Biol. Technol..

[33] Komthong P., Katoh T., Igura N., Shimoda M. (2006). Changes in the odours of apple juice during enzymatic browning. Food Qual Prefer..

[34] Zhu J.C., Niu Y., Xiao Z.B. (2021). Characterization of the key aroma compounds in laoshan green teas by application of odour activity value (OAV), gas chromatography–mass spectrometry-olfactometry (GC–MS–O) and comprehensive two–dimensional gas chromatography mass spectrometry (GC×GC–qMS). Food Chem..

[35] Rudell D.R., Mattinson D.S., Mattheis J.P., Wyllie S.G., Fellman J.K. (2002). Investigations of aroma volatile biosynthesis under anoxic conditions and in different tissues of ‘Redchief Delicious’ apple fruit (*Malus domestica* Borkh.). J. Agric. Food Chem..

[36] Niu Y., Wang R., Xiao Z., Zhu J., Sun X., Wang P. (2019). Characterization of ester odorants of apple juice by gas chromatography-olfactometry, quantitative measurements, odour threshold, aroma intensity and electronic nose. Food Res. Int..

[37] Wu Y., Zhang W., Yu W., Zhao L., Wang S. (2019). Study on the volatile composition of table grapes of three aroma types. LWT–Food Sci. Technol..

[38] Mehinagic E., Royer G., Symoneaux R., Jourjon F., Prost C. (2006). Characterization of odor-active volatiles in apples: Influence of cultivars and maturity stage. J. Agric. Food Chem..

[39] Liu Y.L., Zhang X.J., Zhao Z.Y. (2013). Effects of fruit bagging on anthocyanins, sugars, organic acids and color properties of ‘Granny Smith’ and ‘Golden Delicious’ during fruit maturation. Eur. Food Res. Technol..

